# Enhancing Photocatalytic Degradation Using Cu-CoS_2_ Nanoparticles for Solar-Driven Decolorization of Textile Dye Contaminants in Wastewater

**DOI:** 10.3390/molecules31122152

**Published:** 2026-06-18

**Authors:** Muhammad Idrees, Falak Naz, Uzma Akram, Dilshod Raupov, Utkir Uljayev, Norah A. Albassami, Ahlem Guesmi, Ghulam Abbas Ashraf

**Affiliations:** 1 Department of Chemistry, Bacha Khan University, Charsadda 24420, Khyber Pakhtunkhwa, Pakistan; idrees.ktk0312@gmail.com; 2 Department of Chemistry, Quaid-e-Azam University, Islamabad 45550, Pakistan; uzmakram26@gmail.com; 3 National Research University TIIAME, Kori Niyoziy 39, Tashkent 100000, Uzbekistan; 4 University of Tashkent for Applied Sciences, Str. Gavhar 1, Tashkent 100149, Uzbekistan; 5 Denau Institute of Entrepreneurship and Pedagogy, Denau 190500, Uzbekistan; 6 Chemistry Department, Faculty of Science, King Khalid University, Abha P.O. Box 9004, Saudi Arabia; 7 Chemistry Department, College of Science, Imam Mohammad Ibn Saud Islamic University (IMSIU), Riyadh P.O. Box 5701, Saudi Arabia; 8 Low Dimensional Materials Research Center, Khazar University, Baku AZ1096, Azerbaijan

**Keywords:** sunlight-driven degradation, photocatalytic degradation, Cu-CoS_2_ nanoparticles, wastewater treatment, environmental remediation

## Abstract

Copper cobalt sulfide (Cu-CoS_2_) nanoparticles (NPs) were synthesized via the co-precipitation method in the present study. The synthesized nanoparticles were employed as photocatalysts for the degradation of two hazardous dyes, Eosin B (EB) and Rhodamine B (RB), under sunlight irradiation. The synthesized nanoparticles were characterized using Energy Dispersive X-ray spectroscopy, Scanning Electron Microscopy, UV-Visible spectroscopy, Fourier Transform Infrared spectroscopy, and X-ray Diffraction analysis. The calculated optical band gap of Cu-CoS_2_ was 2.06 eV, while the point of zero charge (PZC) was determined to be 7. The XRD results confirmed the crystalline nature of the Cu-CoS_2_ nanoparticles with an average crystallite size of 28.23 nm. The catalyst exhibited higher photocatalytic degradation efficiency for EB than for RB in single-dye solutions. In contrast, the presence of EB in the binary dye mixture did not significantly influence the degradation of RB. The effects of various operational parameters, including dye concentration, pH, temperature, and catalyst dosage, were systematically investigated. The photocatalytic degradation efficiency of both dyes decreased with increasing initial dye concentration. Optimum degradation conditions for both single and binary dye systems were obtained at dye concentrations of 40:20 μM, pH 5 for EB, pH 9 for RB, and a temperature of 50 °C. The maximum degradation efficiencies achieved in single-dye solutions were 97% for RB and 92% for EB, whereas degradation efficiencies of 98% for RB and 82% for EB were observed in binary dye systems. Furthermore, first-order and second-order kinetic models were applied to evaluate the photodegradation process, and the experimental data showed better agreement with the second-order kinetic model.

## 1. Introduction

The past few decades have witnessed a rapid increase in industrial activities, resulting in the continuous discharge of hazardous organic pollutants into aquatic environments [1]. The presence of these contaminants poses serious threats to aquatic ecosystems as well as human health. Industries such as textiles, pharmaceuticals, tanning, agriculture, chemicals, and cosmetics are major contributors to industrial effluents, thereby causing significant environmental and health concerns [2,3]. These effluents mainly contain toxic organic compounds, heavy metal ions, and synthetic dyes, including Eosin B (EB) and Rhodamine B (RB). Such dyes are considered harmful to humans and other living organisms. EB, a widely used dye in the textile and sanitation industries, exhibits high chemical reactivity and may cause severe environmental impacts [4,5]. Similarly, RB is extensively utilized in various industrial applications and has been associated with skin and eye irritation, as well as gastrointestinal and respiratory disorders. Previous studies have further reported the potential neurotoxic, carcinogenic, and reproductive effects of RB in both humans and animals. Therefore, the proper treatment and controlled disposal of dye-containing industrial effluents into water bodies are of critical importance [6,7,8].

Various physical, chemical, and biological methods have been employed for the removal of these pollutants; however, the persistent and recalcitrant nature of industrial effluents limits the effectiveness of conventional treatment approaches [9]. This highlights the need for advanced and efficient techniques capable of effectively eliminating such contaminants from aquatic environments [10]. Among the available treatment methods, chemical oxidation, adsorption, and photocatalytic degradation have attracted considerable attention because of their ability to remove organic pollutants, including synthetic dyes, from wastewater [11,12]. Among these approaches, photocatalytic degradation is considered one of the most promising and efficient techniques due to its simplicity, cost-effectiveness, and high degradation efficiency [13,14]. Recent studies have demonstrated that transition-metal-based photocatalysts exhibit excellent degradation performance toward organic contaminants through enhanced generation of reactive oxygen species [15,16]. Photocatalysis involves the conversion of solar energy into chemical energy through semiconductor materials, thereby facilitating the degradation of organic pollutants [17,18].

Metal oxide semiconductors such as ZnO, TiO_2_, Fe_2_O_3_, and CuO have been widely investigated as photocatalysts for the degradation of organic contaminants into simpler and less harmful products, including CO_2_ and H_2_O. However, their practical application is limited by their relatively large band gaps in the ultraviolet region [19,20]. Since ultraviolet light constitutes only a small fraction of the solar spectrum, the development of semiconductor materials capable of absorbing visible light has become increasingly important [21,22]. Upon light irradiation, photo-generated electron–hole pairs interact with oxygen and water molecules adsorbed on the surface of the nanoparticles, leading to the formation of highly reactive oxygen species (ROS), such as hydroxyl radicals, superoxide radicals, and singlet oxygen. These reactive species play a vital role in the degradation of organic pollutants without producing harmful secondary residues [23,24].

The unique physical, chemical, and optical properties of semiconductor metal sulfides, along with their narrow band gaps and high charge-carrier mobility, make them promising candidates for the photocatalytic degradation of organic dyes. Metal sulfides such as CuS [25], CoS [26], ZnS [27], and FeS [28] have attracted considerable attention because of their excellent visible-light absorption properties and catalytic performance [29]. The growing interest in metal sulfide semiconductors is mainly attributed to their narrow energy band gaps, which enable their applications in photodetectors, solar cells [30], and batteries [31]. In the present study, copper cobalt sulfide (Cu-CoS_2_) nanoparticles (NPs) were synthesized using the co-precipitation method. The synthesized NPs were used for the photocatalytic degradation of EB and RB under sunlight irradiation. In addition, the effects of various reaction parameters, including irradiation time, catalyst dosage, dye concentration, pH of the reaction medium, and temperature, were systematically optimized.

## 2. Materials and Methods

### 2.1. Reagents

Copper chloride (CuCl_2_·2H_2_O), cobalt chloride (CoCl_2_·6H_2_O), and sodium sulfide (Na_2_S) were purchased from Sigma-Aldrich (St. Louis, MO, USA). EB and RB were obtained from Merck (Darmstadt, Germany). All chemicals used in this study were of analytical grade and were used without further purification.

### 2.2. Instruments

The synthesized nanoparticles were characterized using Fourier transform infrared spectroscopy (FTIR; PerkinElmer Spectrum Two, Waltham, MA, USA), X-ray diffraction (XRD, Rigaku Corporation, Tokyo, Japan), UV-visible spectroscopy (UV-1800, 240 V, Shimadzu Corporation, Nakagyo, Japan), energy-dispersive X-ray spectroscopy (EDX; Oxford Instruments, Abingdon, UK), and scanning electron microscopy (SEM; JEOL Ltd., Tokyo, Japan). FTIR analysis was performed to identify the surface functional groups present in the samples, while XRD analysis was used to investigate the crystalline structure and crystallite size of the synthesized nanoparticles. XRD patterns were recorded using Cu Kα radiation (λ = 1.5406 Å) over a 2θ range of 10–80° at a scan rate of 2° min^−1^. FTIR spectra were acquired in the range of 400–4000 cm^−1^ with a resolution of 4 cm^−1^. UV-visible spectroscopy was employed to determine the dye concentration during photocatalytic experiments. EDX analysis was carried out to evaluate the elemental composition, whereas SEM was used to examine the morphology of the synthesized nanoparticles.

An oven and muffle furnace were utilized for the initial drying and subsequent calcination of the synthesized black precipitate. The centrifugation process was performed using a centrifuge (Daihan Labtech Co., Ltd., Namyangju-si, Korea). Photocatalytic experiments were carried out under natural sunlight at Bacha Khan University, Charsadda (71.830° E, 34.130° N), on clear cloudless days between 11:00 AM and 2:00 PM. The average ambient temperature was 27.5 °C, and the relative humidity was approximately 35%. All experiments were conducted under comparable weather conditions to minimize fluctuations in solar irradiation. This operational window ensured maximum and relatively stable solar flux, maintaining experimental reproducibility across all runs.

### 2.3. Synthesis of Cu-CoS_2_ Nanoparticles

Bimetallic Cu-CoS_2_ (CCS) nanoparticles were synthesized using the chemical co-precipitation method. The synthesis was carried out using copper chloride (CuCl_2_·2H_2_O), cobalt chloride (CoCl_2_·6H_2_O), and sodium sulfide (Na_2_S) as precursor materials. Initially, 0.5 M solutions of CuCl_2_·2H_2_O and CoCl_2_·6H_2_O were mixed and subsequently added dropwise to a 1 M sodium sulfide solution under continuous stirring. The reaction mixture was stirred for 1 h at room temperature, followed by heating at 50 °C for 3 h, during which a black precipitate was formed.

The obtained precipitate was filtered and thoroughly washed with deionized water to remove residual ions and unreacted species. The washed precipitate was then dried in an oven at 80 °C for 2.5 h. Finally, the dried sample was calcined in a muffle furnace at 500 °C for 2 h with a heating ramp rate of 5 °C min^−1^ under ambient air atmosphere. The final product was allowed to cool naturally to room temperature and stored in a clean airtight container for further experimental use.

Although calcination was performed in air, the formation of pure sulfide phase is attributed to the prior complete sulfidation during co-precipitation, while the relatively controlled calcination conditions (moderate temperature and limited duration) help preserve the sulfide phase by minimizing oxidation. This is further supported by XRD and FTIR analyses, which confirm the absence of detectable oxide impurities in the final material.

### 2.4. Photocatalytic Degradation of Dye

The solar photocatalytic degradation experiments were conducted on the campus of Bacha Khan University, Charsadda, Pakistan (71.830° E, 34.130° N). All experiments were performed on clear, cloudless days between 11:00 AM and 2:00 PM to ensure maximum solar irradiation. During this period, the average ambient conditions were recorded as 27.5 °C temperature and 35% relative humidity [32].

Photocatalytic experiments were performed using both single and binary dye systems in an aqueous medium containing EB and RB. Stock solutions (500 µM) of each dye were prepared in deionized water and diluted as required to obtain the desired working concentrations. Binary mixtures were prepared by mixing appropriate volumes of the working solutions to obtain EB:RB concentrations of 30:30 µM (Solution A), 35:25 µM (Solution B), and 40:20 µM (Solution C), corresponding to EB/RB volumes of 50/50 mL, 60/40 mL, and 70/30 mL, respectively.

For each experiment, 0.01 g of Cu-CoS_2_ nanoparticles was added to 100 mL of dye solution, and the suspension was magnetically stirred to ensure uniform dispersion of the catalyst. The reaction mixtures were then exposed to sunlight irradiation. Aliquots (5 mL) were collected at 20 min intervals and centrifuged at 10,000 rpm for 10 min to determine the residual dye concentration. UV-Vis spectroscopy was used to measure the absorbance of the solutions. The percentage degradation of the dye mixture was calculated at the respective maximum absorption wavelengths of EB (517 nm) and RB (554 nm).

To confirm the photocatalytic activity, two control experiments were performed. In the first, the dye solutions containing the catalyst were kept in the dark for 1 h to evaluate adsorption–desorption equilibrium, and no significant degradation was observed. In the second, photolysis experiments were conducted by exposing catalyst-free dye solutions to natural sunlight for 1 h, which resulted in negligible self-decolorization, with degradation efficiencies of 9.0% for EB and 4.6% for RB. Based on these control experiments, the solar-driven photocatalytic degradation was evaluated in the presence of Cu-CoS_2_ nanoparticles, and the degradation efficiency was calculated using Equation (1).

(1) $$\%Degradation=\frac{C_{0}-C}{C_{0}}\times 100$$

Here, $C_{0}$ means the initial concentration of the dye before photocatalytic degradation begins (at time t = 0). 

### 2.5. Statistical Analysis and Response Surface Methodology (RSM) Modeling

To mathematically correlate the independent operational parameters with the experimental responses, the complete dataset of input variables and their corresponding outputs was analyzed using multiple regression analysis. The statistical modeling was performed using the Design-Expert software package (Version 11) [33]. A second-order polynomial (quadratic) response surface methodology was employed to describe the empirical relationships, evaluate interaction effects, and predict the response variables, as expressed in Equation (2) [34].

(2) $$Y=f\left(x_{1},x_{2},\ldots ,x_{n}\right)=\beta_{0}+\sum_{i=1}^{n}\beta_{i}x_{i}+\sum_{i=1}^{n}\beta_{ii}x_{i}^{2}+\sum_{i=1}^{n-1}\sum_{j=i+1}^{n}\beta_{ij}x_{i}x_{j}+\varepsilon$$

Here, Y represents the predicted response variable, n denotes the number of independent factors studied, and ε represents the random experimental error or residual term, which is generally assumed to be negligible within the RSM framework. The regression coefficients β_0_, β_i_, and β_ij_ correspond to the intercept, linear effects, quadratic contributions, and interaction effects, respectively.

Analysis of variance (ANOVA) was performed to evaluate the statistical significance of the developed empirical models, determine the contribution of individual terms, and assess the strength of the relationships between independent variables and the response. The goodness-of-fit of the regression models was assessed using the coefficient of determination (R^2^).

After model validation, a numerical optimization approach was applied to identify the optimal set of operating conditions for maximizing dye degradation efficiency. For this purpose, a desirability function approach was employed. This method provides a robust framework for multi-objective optimization by transforming each predicted response into a dimensionless desirability value. In this study, a “larger-the-better” criterion was applied, as expressed in Equation (3), to maximize degradation efficiency.

(3) $$d_{i}=\left\{\begin{array}{cc} 0 & \hat{y_{i}}<L_{i}\\ \frac{\hat{y_{i}}-L_{i}}{T_{i}-L_{i}} & L_{i}\leq \hat{y_{i}}\leq T_{i}\\ 1.0 & T_{i}<\hat{y_{i}} \end{array}\right.$$

$$D={\left(d_{1}\times d_{2}\times \ldots \times d_{k}\right)}^{\frac{1}{k}},\;0\leq D\leq 1$$

Here, L_i_ and T_i_ represent the lower bound and target maximum value of the ith response within the investigated experimental domain, respectively, while k denotes the total number of responses considered for optimization. The optimal operating conditions were selected based on the highest global desirability index, with values approaching unity indicating the best overall solution.

## 3. Results and Discussion

### 3.1. Scanning Electron Microscopy (SEM) Analysis

**Figure 1:**
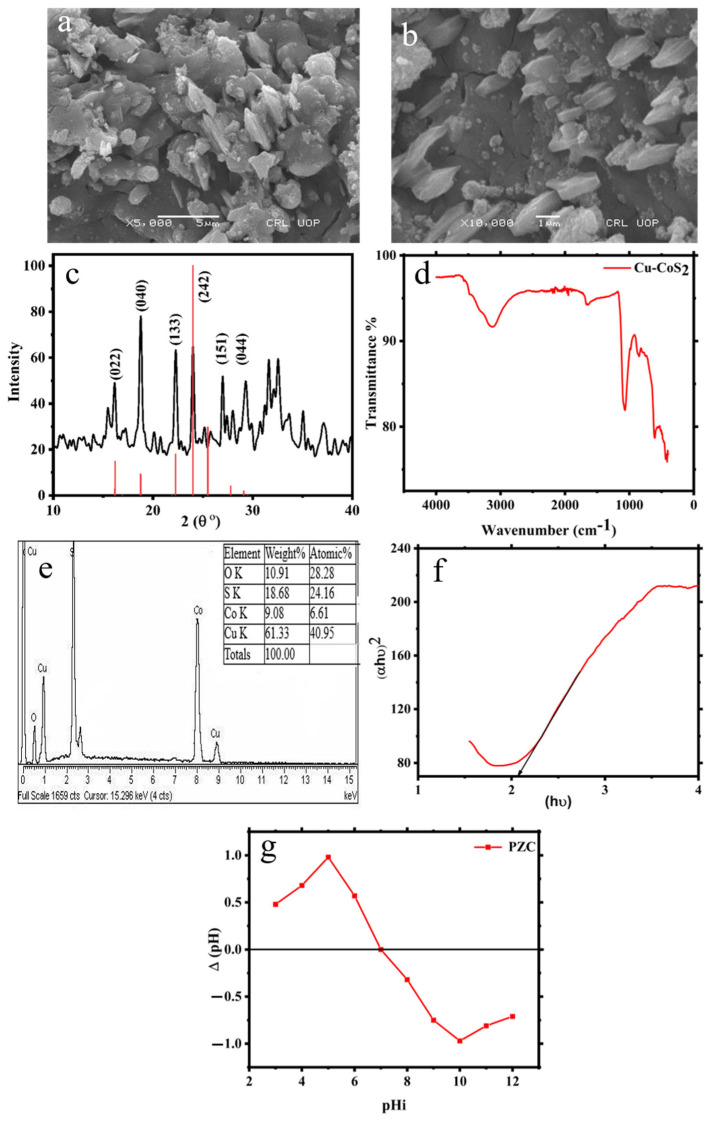
Characterization of synthesized Cu-CoS_2_ nanoparticles. (**a**,**b**) SEM micrographs at different magnifications showing the plate-like morphology and microplate structures of the synthesized Cu-CoS_2_; (**c**) powder XRD pattern indexed to the cubic phase (JCPDS No. 96-153-5419), indicating an average crystallite size of 28.23 nm calculated using the Scherrer equation; (**d**) FTIR spectra highlighting characteristic metal-sulfur (M-S) vibrational bands, confirming successful formation of the Cu-CoS_2_ framework; (**e**) EDX spectrum illustrating the elemental composition and high purity of the material; (**f**) Tauc plot derived from UV-Vis absorbance data revealing an optical band gap of 2.06 eV; (**g**) PZC plot determined by the solid addition method, indicating a pH_p_zc value of 7.

The surface morphology, particle topography, and microstructural features of the synthesized cubic Cu-CoS_2_ catalyst were examined using SEM. Representative SEM micrographs at different magnifications are presented in Figure 1a,b. The images indicate that the material predominantly consists of well-defined, discrete plate-like microstructures rather than spherical nanoparticles. At higher magnification (Figure 1b), these plate-like structures exhibit smooth surfaces with well-defined edges. A moderate degree of particle aggregation is also observed, leading to the formation of interconnected networks with varying orientations.

To quantitatively assess the size distribution of the synthesized material, statistical analysis was performed using ImageJ software (version 1.54h). The results show that the Cu-CoS_2_ microplates have an average length of 1.45 µm.

### 3.2. X-Ray Diffraction (XRD) Analysis

XRD analysis was used to investigate the crystalline structure of the synthesized Cu-CoS_2_ material [35]. The XRD pattern is shown in Figure 1c. The diffraction peaks were indexed and matched with standard reference data using X’Pert HighScore Plus software (version 3.0; PANalytical B.V., Almelo, Netherlands). The results confirm a cubic crystal structure for the synthesized material, consistent with JCPDS card No. 96-153-5419 [36].

The diffraction pattern exhibits well-defined Bragg peaks at 2θ values of approximately 16.2°, 18.8°, 22.26°, 24.03°, 27.02°, and 29.32°. These reflections correspond to the (022), (040), (133), (242), (151), and (044) lattice planes, respectively. In Figure 1c, the black line represents the experimental XRD pattern, while the red vertical lines indicate the standard reference positions. Minor additional peaks may be attributed to trace impurities. All of the indexed diffraction peaks were explicitly utilized to calculate the average crystallite size of the synthesized nanostructures using the Debye-Scherrer equation. (Equation (4)).

(4) $$D=\frac{K\lambda }{\beta \cos\theta }$$

Here, D represents the average crystallite size of the Cu-CoS_2_ nanoparticles, K is the dimensionless Scherrer shape factor (typically 0.94), λ is the wavelength of the X-ray source, β is the full width at half maximum of the diffraction peak expressed in radians, and θ is the Bragg diffraction angle. Using this equation, the average crystallite size of the synthesized material was calculated to be 28.23 nm.

### 3.3. FT-IR Analysis

FTIR spectroscopy was performed in the range of 4000–400 cm^−1^ to investigate the surface chemical composition and bonding characteristics of the synthesized Cu-CoS_2_ microplates (Figure 1d). The spectrum shows several characteristic absorption bands, confirming the formation of the transition metal sulfide framework. A broad absorption band observed between 3100–3400 cm^−1^ is attributed to O-H stretching vibrations arising from surface hydroxyl groups and adsorbed moisture [37]. The sharp band at 1060 cm^−1^ is assigned to Co=S stretching vibrations [38]. The peaks at approximately 605 cm^−1^ and 417 cm^−1^ are attributed to metal-sulfur (M-S) stretching and bending vibrations, respectively, confirming the successful formation of the Cu-CoS_2_ structure [39].

### 3.4. Energy Dispersive X-Ray (EDX) Analysis

EDX is commonly used to determine the elemental composition of synthesized materials [40]. To confirm the formation of Cu-CoS_2_ nanoparticles, EDX analysis was performed, and the spectrum is shown in Figure 1e. The results reveal the presence of all constituent elements with distinct peaks corresponding to Cu, Co, S, and O.

In Cu-CoS_2_, the oxygen content was 28.28% (atomic) and 10.91% (weight), sulfur was 24.16% (atomic) and 18.68% (weight), cobalt was 6.61% (atomic) and 9.08% (weight), and copper was 61.33% (atomic) and 40.54% (weight). No additional peaks corresponding to other elements were detected, confirming the presence of Cu, Co and S as major constituents, together with minor surface oxygen species.

### 3.5. Optical Band Gap of Cu-CoS_2_

The optical absorption characteristics and electronic band structure of the synthesized Cu-CoS_2_ microplates were investigated using UV-Visible spectrophotometry across a wavelength range of 200 to 800 nm (Figure 1e). A small quantity of the prepared sample was uniformly dispersed in absolute ethanol to record its characteristic electronic transitions. To determine the precise optical bandgap (E_g_) of the prepared catalyst, the collected absorbance data were converted and analyzed using the classic Tauc relation (Equation (5)):

(5) $${\left(\alpha h\nu \right)}^{n}=A\left(h\nu -E_{g}\right)$$

where α is the optical absorption coefficient, h represents the incident photon energy, A is a material-dependent proportionality constant, E_g_ is the optical bandgap, and the exponent n determines the nature of the electronic transition. The linear extrapolation of the ${\left(\alpha h\nu \right)}^{2}$ versus photon energy (hv) plot yielded an optical bandgap value of 2.06 eV for the Cu-CoS_2_, as illustrated in the Tauc plot (Figure 1f). This moderate, visible-light-responsive bandgap confirms that the synthesized catalyst possesses favourable electronic architecture to maximize solar photon harvesting, effectively generating the electron-hole (e^−^/h^+^) pairs required to drive the rapid degradation of both EB and RB dyes under natural sunlight.

### 3.6. Point of Zero Charges (PZC)

The solid addition method was used to determine the PZC of the synthesized particles. Eight separate 0.1 M KNO_2_ solutions were prepared, and the initial pH (pH_i_) was adjusted in the range of 3–12 using 0.01 M HCl or 0.01 M NaOH. Cu-CoS_2_ (18 mg) was added to each solution, and the suspensions were stirred at 60 rpm for 24 h. After equilibration, the solutions were filtered and the final pH values were recorded.

The PZC was determined by plotting the initial pH against the change in pH (ΔpH). The point where ΔpH = 0 was taken as the PZC, which was found to be 7. Figure 1g shows the PZC value of the Cu-CoS_2_ nanoparticles.

### 3.7. Optimization of Dye Degradation

The photocatalytic efficiency of the Cu-CoS_2_ nanoparticles is governed by the generation, migration, and utilization of photo-excited charge carriers. Upon irradiation with solar light, photons possessing energy equal to or greater than the bandgap energy of the Cu-CoS_2_ semiconductor are absorbed, leading to the excitation of electrons e^−^ from the valence band (VB) to the conduction band (CB) [41]. This process results in the formation of electron-hole e^−^/h^+^ pairs (Equation (6)). These photogenerated charge carriers migrate to the catalyst surface, where they initiate the degradation of organic pollutants through two primary pathways. In the direct pathway, the highly oxidative holes in the VB and reductive electrons in the CB react directly with the adsorbed dye molecules (RB and EB), destabilizing their complex aromatic structures. In the indirect pathway, the surface-migrated charge carriers interact with ambient molecules to produce ROS. Specifically, holes in the VB react with adsorbed water molecules or hydroxyl ions to generate hydroxyl radicals, which are powerful, non-selective oxidizing agents (Equations (7) and (8)). Concurrently, electrons in the CB reduce dissolved oxygen molecules to form superoxide radicals (Equation (9)). These ROS facilitate the successive oxidative cleavage of the dye’s chromophore groups, breaking them down into smaller intermediate fragments. Ultimately, these intermediates undergo complete mineralization into non-toxic end-products, primarily CO_2_, H_2_O and inorganic ions such as sulfates and nitrates, depending on the elemental composition of the initial contaminant (Equation (10)) [42].

(6) $${\mathrm{Cu}\text{-}\mathrm{CoS}}_{2}+h\nu \to {\mathrm{Cu}\text{-}\mathrm{CoS}}_{2}\left(e_{CB}^{-}+h_{VB}^{+}\right)$$

(7) $$h_{VB}^{+}+H_{2}O\to \cdot OH+H^{+}$$

(8) $$h_{VB}^{+}+OH^{-}\to \cdot OH$$

(9) $$e_{CB}^{-}+O_{2}\to O_{2}^{\cdot -}$$

(10) $$\mathrm{Dye}+\cdot OH/O_{2}^{\cdot -}\to \mathrm{Intermediates}\to CO_{2}+H_{2}O+\mathrm{Inorganic}\;\mathrm{ions}$$

#### 3.7.1. Effect of Dye Concentration

**Figure 2:**
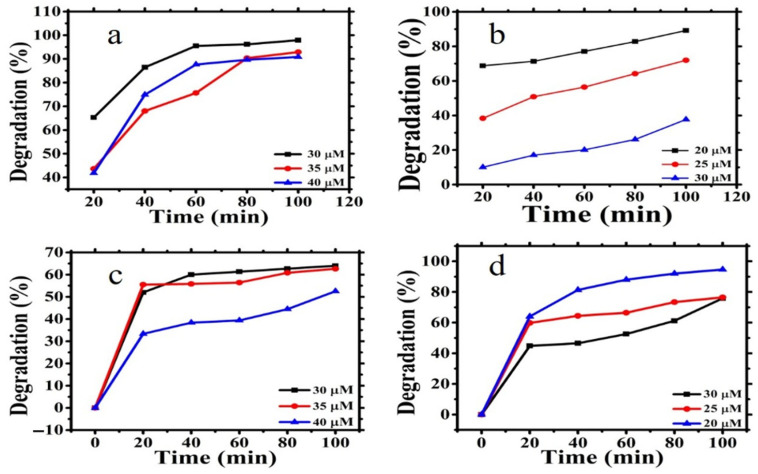
Photocatalytic degradation of EB and RB dyes using Cu-CoS_2_. (**a**) Effect of dye concentration on the degradation of single EB dye (30, 35, and 40 μM); (**b**) effect of dye concentration on the degradation of single RB dye (20, 25, and 30 μM); (**c**,**d**) percentage degradation of EB and RB in binary mixtures A, B, and C with varying concentration ratios and volumes. Experimental conditions: catalyst dose = 0.01 g; temperature = 50 °C.

The effect of dye concentration on degradation efficiency was evaluated at 50 °C using a catalyst dose of 0.01 g. For the single-dye system, EB degradation decreased with increasing concentration: 97.91% at 30 μM, 92.89% at 35 μM, and 90.83% at 40 μM using Cu-CoS_2_ (Figure 2a). In the case of RB, the degradation efficiencies were 89.17% at 20 μM, 71.98% at 25 μM, and 37.68% at 30 μM (Figure 2b).

Three binary dye mixtures (A, B, and C) were prepared with different EB:RB ratios. Mixture A (30:30 μM; 50:50 mL) showed degradation efficiencies of 64% for EB and 75.86% for RB. Mixture B (35:25 μM; 60:40 mL) resulted in 62.63% degradation for EB and 76.50% for RB. Mixture C (40:20 μM; 70:30 mL) exhibited 52.52% degradation for EB and 94.66% for RB (Figure 2c,d).

In the binary system, when the concentration of EB was higher than that of RB, the degradation efficiency of EB decreased, whereas that of RB increased. In contrast, in single-dye systems, EB exhibited higher degradation efficiency than RB. The decrease in degradation efficiency with increasing dye concentration can be attributed to the higher adsorption of organic molecules on the catalyst surface, which limits photon penetration and reduces the generation of reactive hydroxyl species, ultimately lowering the overall degradation rate.

#### 3.7.2. Effect of Catalyst Dose

**Figure 3:**
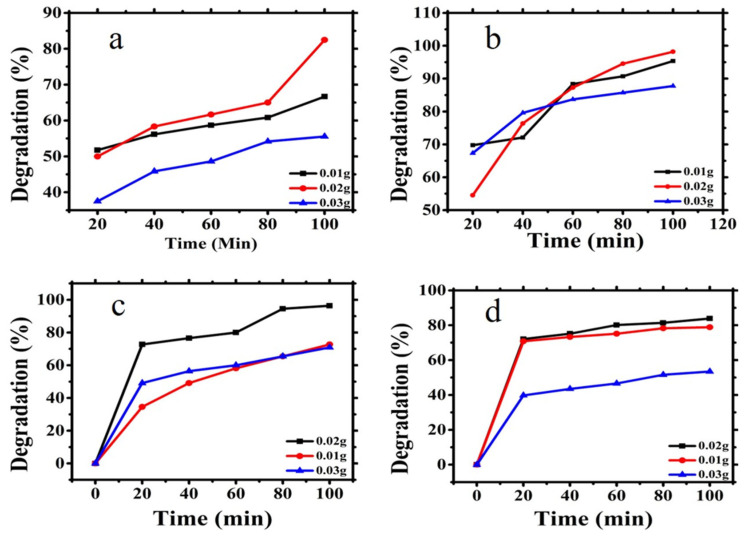
Effect of Cu-CoS_2_ catalyst dosage on the photodegradation efficiency of EB and RB dyes. (**a**,**b**) Binary mixture: Photodegradation of an EB:RB mixture (40:20 μM) at 50 °C under 100 min of irradiation. The degradation performance varied with catalyst dosage: at 0.01 g, the efficiencies were 66.66% (EB) and 95.34% (RB); at 0.02 g, 55.55% (EB) and 98.18% (RB); and at 0.03 g, 98.18% (EB) and 87.75% (RB). (**c**) Single dye (EB): degradation efficiencies of 72.7%, 96.36%, and 70.90% at 0.01 g, 0.02 g, and 0.03 g, respectively. (**d**) Single dye (RB): degradation efficiencies of 53.41%, 83.85%, and 78.88% at the same catalyst dosages.

The effect of catalyst dosage on the photodegradation of the binary EB:RB (40:20 μM) mixture was investigated at different catalyst doses (0.01, 0.02, and 0.03 g) under constant irradiation for 100 min at 50 °C. It was observed that the degradation efficiency varied non-linearly with catalyst loading. At 0.01 g, the degradation efficiencies of EB and RB were 66.66% and 95.34%, respectively. At 0.02 g, the corresponding values were 55.55% and 98.18%, while at 0.03 g, the efficiencies were 98.18% for EB and 87.75% for RB (Figure 3a,b).

In the single-dye system, a similar trend was observed, where degradation initially increased with catalyst dose and then decreased beyond an optimum value. For EB, degradation efficiencies were 72.72% at 0.01 g, 96.36% at 0.02 g, and 70.90% at 0.03 g (Figure 3c). For RB, the corresponding values were 53.41%, 83.85%, and 78.88%, respectively (Figure 3d).

The overall trend indicates that increasing catalyst dosage initially enhances dye degradation due to the availability of more active sites. However, beyond an optimal loading, the degradation efficiency decreases due to increased turbidity of the reaction mixture and reduced light penetration, which limits photon access to active sites and consequently decreases the generation of reactive hydroxyl radicals.

#### 3.7.3. Effect of pH

**Figure 4:**
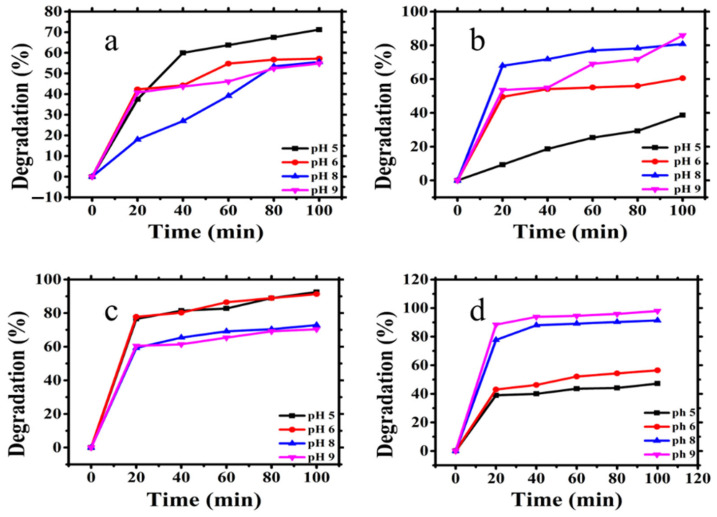
pH-dependent photodegradation of EB and RB dyes by Cu-CoS_2_. The plots show degradation behavior in binary (**a**,**b**) and single-dye (**c**,**d**) systems. EB exhibits maximum degradation under acidic conditions (pH 5) due to favorable electrostatic attraction with the positively charged catalyst surface. In contrast, RB shows highest degradation in alkaline medium, reaching 98.49% at pH 9, attributed to enhanced interaction with the negatively charged catalyst surface. Experimental conditions: 0.01 g catalyst, 50 °C, 100 min irradiation.

The effect of pH on the photodegradation of the binary EB:RB (40:20 μM) mixture was investigated at different pH values (5, 6, 8, and 9) under constant conditions of catalyst dose (0.01 g), temperature (50 °C), and irradiation time (100 min). The results indicate a strong pH-dependent behavior. At pH 5, the degradation efficiencies of EB and RB were 71.25% and 38.66%, respectively. At pH 6, the corresponding values were 57.21% and 60.55%. At pH 8, EB and RB showed degradation efficiencies of 55.55% and 80.76%, while at pH 9, the values were 54.85% and 85.91% using Cu-CoS_2_ (Figure 4a,b).

In the single-dye systems, a similar trend was observed. Under acidic conditions (pH < 7), EB exhibited higher degradation than RB, whereas under alkaline conditions (pH > 7), RB showed higher degradation efficiency. For EB, the degradation efficiencies were 92.59% (pH 5), 91.35% (pH 6), 72.83% (pH 8), and 70.37% (pH 9) (Figure 4c). For RB, the corresponding values were 37.68% (pH 5), 56.46% (pH 6), 92.46% (pH 8), and 98.49% (pH 9) (Figure 4d).

The observed pH-dependent behavior can be attributed to the surface charge characteristics of the Cu-CoS_2_ nanoparticles. Below the point of zero charge (pH_p_zc), the catalyst surface is positively charged, whereas above pH_p_zc it becomes negatively charged. Consequently, electrostatic interactions govern dye adsorption and degradation: cationic dyes are more efficiently degraded in alkaline media, while anionic dyes show higher degradation efficiency in acidic conditions, thereby enhancing overall photocatalytic performance.

#### 3.7.4. Effect of Temperature

**Figure 5:**
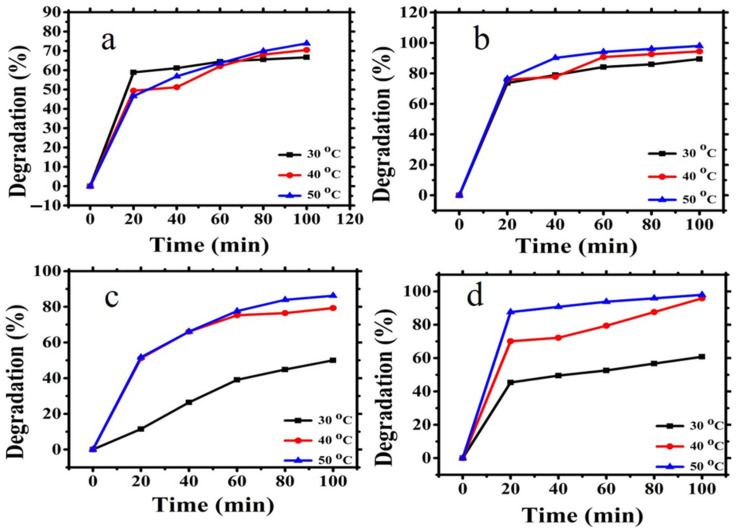
Influence of reaction temperature on dye degradation efficiency using Cu-CoS_2_ catalyst. (**a**,**b**) Binary dye mixture: degradation efficiencies of EB and RB in a combined system. Maximum degradation (72.15% for EB and 98.03% for RB) was achieved at 50 °C, compared with 66.66% and 89.47% at 30 °C, respectively. (**c**) Single dye (EB): degradation efficiency increased from 50% at 30 °C to 86.20% at 50 °C. (**d**) Single dye (RB): degradation efficiency increased from 60.8% at 30 °C to 97.93% at 50 °C. Experimental conditions: constant catalyst dosage and initial dye concentration across temperatures of 30 °C, 40 °C, and 50 °C.

The effect of temperature on the photodegradation of the binary EB:RB mixture was investigated at different temperatures (30 °C, 40 °C, and 50 °C) under constant catalyst dose and initial dye concentration. The results indicate that dye degradation increases with increasing temperature, with the highest efficiency observed at 50 °C. At 30 °C, the degradation efficiencies of EB and RB were 66.66% and 89.47%, respectively. At 40 °C, the corresponding values increased to 70.48% and 94.44%, while at 50 °C they reached 72.15% and 98.03% using Cu-CoS_2_ (Figure 5a,b).

In the single-dye system, a similar trend was observed, where higher temperatures enhanced degradation efficiency for both dyes. For EB, degradation efficiencies were 50% at 30 °C, 79.31% at 40 °C, and 86.20% at 50 °C (Figure 5c). For RB, the corresponding values were 60.8%, 95.87%, and 97.93%, respectively (Figure 5d).

The enhanced photocatalytic performance at elevated temperatures can be attributed to increased kinetic energy of dye molecules, which enhances diffusion toward the Cu-CoS_2_ catalyst surface and increases the frequency of effective collisions with photogenerated reactive species (OH^•^ and O_2_^•−^). Additionally, the degradation process is generally endothermic in nature, and higher temperatures provide sufficient energy to overcome activation barriers associated with the oxidative breakdown of complex dye structures [43,44].

### 3.8. Photocatalytic Kinetics

The photodegradation of anionic and cationic dyes was investigated in both single and binary aqueous systems using Cu-CoS_2_ nanoparticles as the catalyst. The degradation behavior of EB (anionic) and RB (cationic) was evaluated in individual dye solutions as well as in EB-RB binary mixtures.

For both single and binary systems, the photodegradation kinetics were analyzed using first-order and second-order kinetic models, as expressed in Equations (11) and (12).

(11) $$ln\frac{C_{0}}{Ct}=k_{app}\cdot t$$

(12) $$\frac{1}{C}-\frac{1}{C_{0}}=k_{app}\cdot t$$

where C_0_ is the initial concentration, C is the real-time dye concentration at time t, t is time, and kapp is the apparent rate constant.

**Figure 6:**
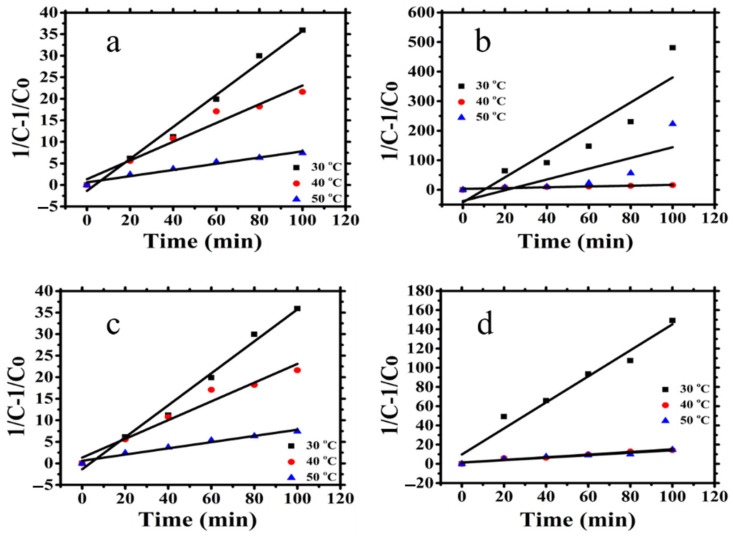
Kinetic modeling of dye photodegradation. Second-order kinetic analysis of EB and RB degradation: (**a**) second-order plot for EB degradation; (**b**) second-order plot for RB degradation; (**c**) second-order plot for EB degradation under modified conditions; (**d**) second-order plot for RB degradation under modified conditions. The solid lines represent the linear regression fit to the experimental data points.

Photocatalytic degradation kinetics were analyzed using both first-order and second-order models at different temperatures. The first-order kinetic fitting is shown in Figure S1, while the second-order kinetic analysis for the temperature study is presented in Figure 6a–d. The corresponding R^2^ and rate constants are provided in Table 1 and Table S1.

The photocatalytic degradation kinetics were analyzed using both first-order and second-order kinetic models. The results indicate that the second-order kinetic model exhibited higher correlation coefficients (R^2^ values) and provided a better fit to the experimental data than the first-order model in both single and binary dye systems. Although the Langmuir-Hinshelwood model and pseudo-first-order kinetics are commonly employed in photocatalytic degradation studies, deviations from first-order behavior can occur due to complex adsorption–desorption equilibria, dye-dye interactions, surface heterogeneity, and competitive utilization of active sites, particularly in multi-component systems. Therefore, under the investigated experimental conditions, the second-order kinetic model was considered more appropriate for describing the degradation behavior of EB and RB. Similar observations have been reported for photocatalytic degradation systems in previous studies [45,46].

Additionally, the degradation rate of EB in the binary mixture shows no significant difference compared to its degradation in the single-dye system. These findings indicate that the presence of EB does not significantly affect the degradation behavior of RB in the binary solution.

### 3.9. Arrhenius Equation

The activation energy for the degradation of dyes was determined by using the Arrhenius equation. The following Equation (13) was used:

(13) K=Ae−EaRT

The logarithmic form of this Equation (14) is given as;

(14) $$lnK=lnA-\frac{Ea}{RT}$$

**Figure 7:**
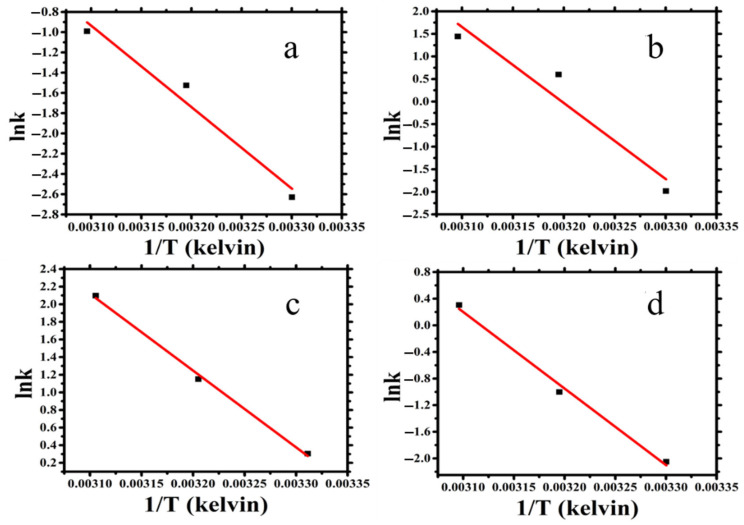
Arrhenius plots for the degradation of dyes: (**a**) Arrhenius plot for Dye A degradation; (**b**) Arrhenius plot for Dye B degradation; (**c**) Arrhenius plot for Dye C degradation; and (**d**) Arrhenius plot for Dye D degradation. All regression lines show strong negative correlation.

Here, k is the rate constant of the reaction, A is the Arrhenius pre-exponential factor, E_a_ is the activation energy, and R is the universal gas constant. A linear relationship was obtained when ln k was plotted against 1/T, yielding a straight line with a negative slope (Figure 7a–d and Figure S2). The activation energy values calculated for the degradation of single and binary dye systems are presented in Table 2 and Figure S2.

### 3.10. Mechanistic Insights into Binary Dye Degradation

The photocatalytic performance in a multi-component system is significantly influenced by the interaction between different dye molecules and the catalyst surface [47]. In this study, the presence of EB was found to have a negligible inhibitory effect on the degradation of RB. This behavior can be explained through several physicochemical mechanisms. The Cu-CoS_2_ catalyst surface possesses a distribution of active sites with varying electronic densities. Because RB is a cationic dye and EB is anionic, they do not compete for the same electrostatic adsorption sites. As noted in studies of cellulose-based bioadsorbents, dyes with opposite charges are governed by different electrostatic attraction forces [48]. RB molecules are attracted to negatively charged surface regions, while EB molecules preferentially occupy positively charged sites. This site-specific adsorption prevents the site-blocking effect typically observed in single-charge binary systems. In binary systems involving complex aromatic structures, synergistic effects can enhance co-adsorption. The literature suggests that at specific concentrations, the interaction between different dyes and the catalyst surface involves a dynamic balance between competition and synergism [49]. In our system, the coexistence of RB and EB may facilitate intermolecular π-π stacking and hydrogen bonding, which assist in the formation of a dye layer on the catalyst surface, thereby maintaining a high concentration of reactants near the generated ROS.

The photocatalytic performance of the synthesized Cu-CoS_2_ nanoparticles was further compared with previously reported photocatalysts in the literature, as summarized in Table 3, highlighting its competitive and superior degradation efficiency under natural sunlight conditions.

### 3.11. Recyclability and Reusability of the Cu-CoS_2_ Photocatalyst

**Figure 8:**
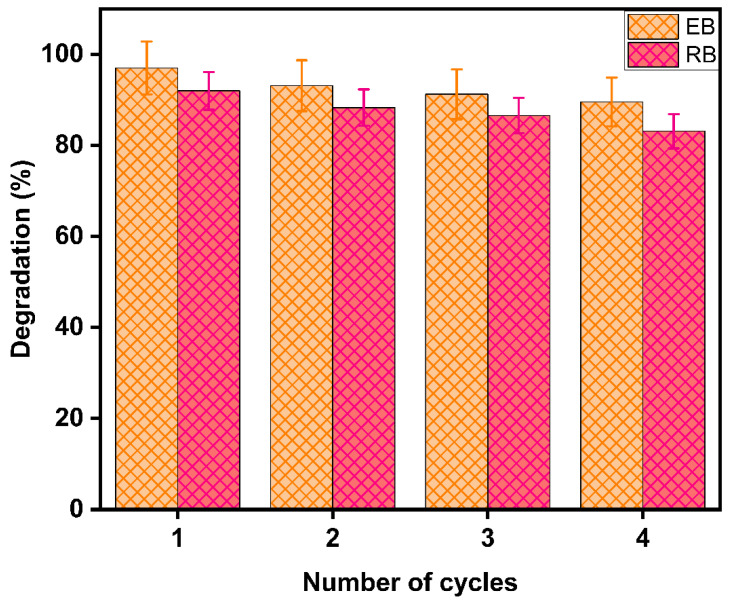
Solar-driven photocatalytic recyclability and long-term stability of Cu-CoS_2_ nanoparticles for the degradation of EB and RB over four consecutive catalytic cycles under optimized conditions.

The stability and reusability of a photocatalyst are critical factors for its practical application in industrial wastewater treatment [50]. Therefore, the photocatalytic activity of the recycled Cu-CoS_2_ nanoparticles was systematically evaluated across three successive cycles under optimized experimental conditions. After each run, the spent photocatalyst was recovered, washed several times thoroughly with distilled water to remove residual adsorbed dye molecules, dried completely, and reused in the subsequent experiment. The recycled catalyst continued to exhibit highly efficient performance for both target contaminants; however, a minor decrease in photocatalytic activity was observed compared to the pristine material. As illustrated in Figure 8, for EB, the degradation rate shifted from an initial 97.00% using the pristine catalyst to 93.12% in the second cycle (first reuse), dropping to 91.24% and 89.52% in the third and fourth cycles, respectively. A similar resilient trend was observed for RB, where the degradation efficiency began at 92.00%, and measured 88.32%, 86.54%, and 83.11% during the subsequent runs. This progressive reduction in degradation efficiency over multiple runs can be attributed to the inevitable occupancy or blockage of a fraction of active catalytic surface sites by persistent dye degradation intermediates, which limits the available surface area for subsequent photon absorption and radical generation.

**Table 3:** Comparison of the current work with literature.

Catalyst	Dye	Light Source	Time (min)	Degradation (%)	Reference
K-doped CdO nanoparticles	EB	Visible Light	140	90.0%	[51]
Co-Fe_2_O_3_	EB	Visible Light	140	82.0%	[52]
BiVO_4_	EB	UV-Visible	120	55%	[53]
Mo_2_C	EB	visible light	60	96.4%	[54]
ZnS	EB	Visible Light	300 min	63%	[55]
TiO_2_	RB	Solar Light	90 min	76.7%	[56]
TiO_2_/ZrO_2_	RB	Visible Light	270 min	90.5%	[57]
ZnO	RB	UV light	160	95.41%	[58]
SnO_2_/Bi_2_S_3_-Bi25	RB	Simulated Sunlight	180	80.0%	[59]
ZnO/MoO_3_	RB	visible light LEDs	90	96.9%	[60]
Cu-CoS_2_	EB	Natural Sunlight	100	92%	Present Study
Cu-CoS_2_	RB	Natural Sunlight	100	97%	Present Study

### 3.12. RSM Modelling and Analysis of EB and RB Degradation

RSM was employed to evaluate and optimize the degradation of EB and RB using the synthesized Cu-CoS_2_ photocatalyst. The effects of five independent operating variables, namely contact time (A), dye concentration (B), Cu-CoS_2_ dose (C), pH (D), and temperature (E), on dye degradation efficiency were systematically investigated using a central composite design. Quadratic regression analysis was applied to establish statistically significant mathematical models correlating the experimental variables with the degradation efficiency of EB and RB. The developed regression equations in terms of actual factors for EB and RB degradation are represented by Equations (15) and (16), respectively.

(15) $$\begin{array}{cc} Y_{\left(EB\right)}=310.59699 & -3.59163A+3.90054B-14747.24848C+81.68665D\\ & -17.03273E-0.005815AB-0.688990AD+0.263363AE\\ & -14.15915BC-0.016583BE+415.02760CD\\ & +326.26234CE-0.421990DE-0.017217A^{2}-0.031277B^{2}\\ & -8351.11489C^{2}-2.16532D^{2}-0.014869E^{2} \end{array}$$

(16) $$\begin{array}{cc} Y_{\left(RB\right)}=92.49536 & +1.07303A-21.70803B+1471.02130C+10.08332D\\ & +6.63964E+0.158499AB-19.82663AC-0.113155AD\\ & -0.062110AE+417.97393BC+0.238063BD\\ & +0.107940BE+317.85882CD-234.52279CE\\ & +0.022256DE-0.007570A^{2}-0.069951B^{2}\\ & -73893.63383C^{2}-0.602850D^{2}-0.002762E^{2} \end{array}$$

Here, Y(EB) and Y(RB) represent the degradation efficiencies (%) of EB and RB, respectively. The regression equations describe the linear, interaction, and quadratic effects of the studied variables on dye degradation. Positive coefficients indicate synergistic effects, whereas negative coefficients indicate inhibitory effects.

The ANOVA results for the reduced quadratic model describing EB degradation are presented in Table S3 (Supporting Information). The model exhibits a high F-value of 183.44 with a *p*-value < 0.0001, indicating strong statistical significance. This implies that there is only a 0.01% probability that such a large F-value could arise due to random experimental error. The validity of the model is further supported by the lack-of-fit test, which yields an F-value of 0.83 and a *p*-value of 0.6036, indicating that the lack of fit is not significant relative to the pure error. This confirms a good agreement between the model and the experimental data.

Similarly, the ANOVA results for the full quadratic model of RB degradation demonstrate strong statistical significance, with an F-value of 100.00 and *p* < 0.0001 (Table S4). As with the EB system, there is only a 0.01% probability that this result is due to random variation. The lack-of-fit F-value for the RB model is 2.91 with a *p*-value of 0.0692, indicating non-significance and further validating the model.

For both systems, the high R^2^ and adjusted R^2^ values indicate that the models explain more than 98% of the total variance in dye degradation. The coefficient of variation (C.V.%) is low for both EB (1.40%) and RB (2.95%), demonstrating good reproducibility and experimental precision across independent runs.

### 3.13. Evaluation of RSM Interaction Profiles for EB and RB Degradation

The individual and synergistic effects of the key operational parameters on the photocatalytic degradation of EB and RB were systematically evaluated. The corresponding 2D contour plots and 3D response surface plots were analyzed, where blue regions represent minimum degradation efficiency and red regions indicate maximum response values.

**Figure 9:**
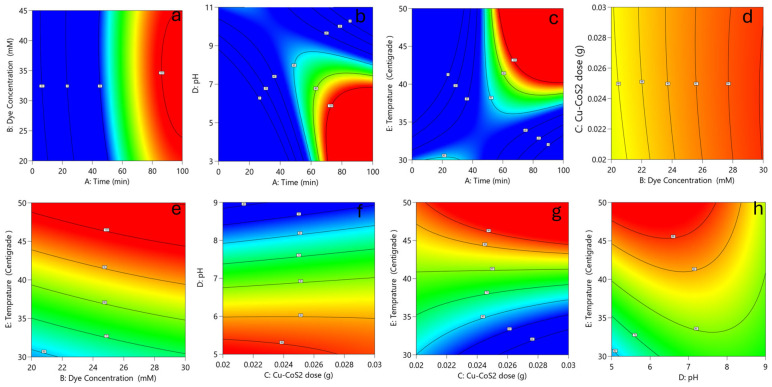
(**a**–**h**) 2D contour plots of EB removal.

Figure 9a–c illustrate the interactive effects of irradiation time with initial dye concentration, pH, and reaction temperature on EB removal, respectively. The results show that degradation efficiency increases with increasing irradiation time. Maximum EB degradation was achieved at lower initial dye concentrations (25–30 μM) (Figure 9a). In addition, lower pH values enhanced degradation efficiency (Figure 9b), while higher reaction temperatures further improved photocatalytic performance across all conditions. A synergistic effect was also observed, where lower pH combined with intermediate catalyst dosage resulted in enhanced degradation efficiency.

**Figure 10:**
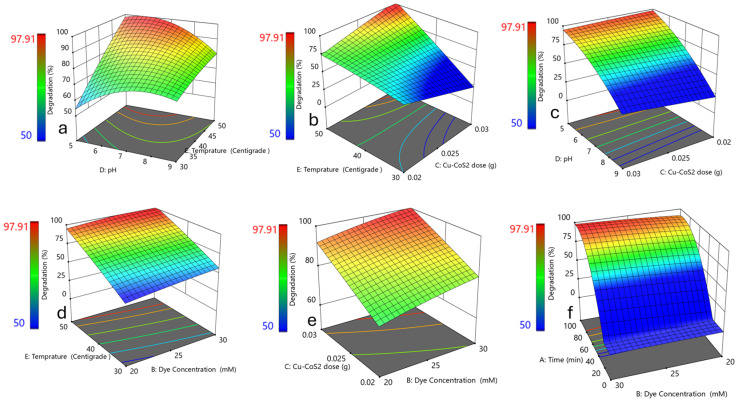
(**a**–**f**) 3D response surface plots of EB removal.

**Figure 11:**
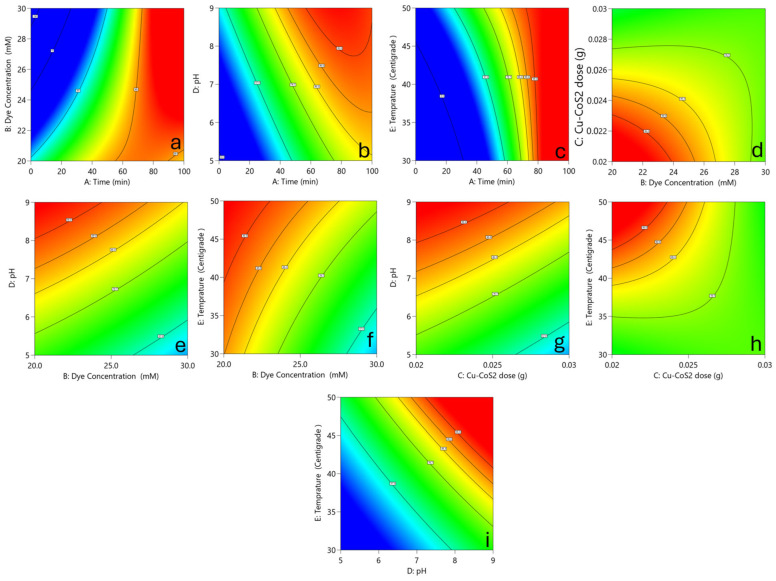
(**a**–**i**) 2D contour plots of RB removal.

**Figure 12:**
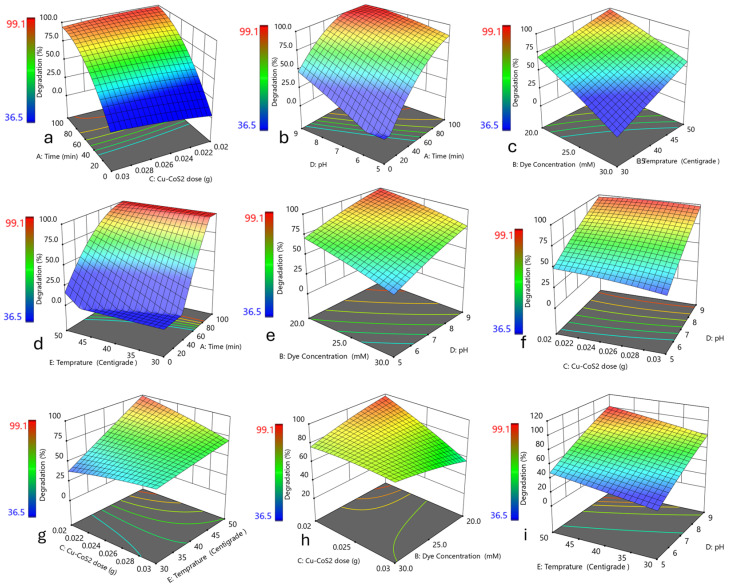
(**a**–**i**) 3D response surface plots of RB removal.

Overall, the 3D response surface plots confirm that optimal EB removal is achieved under conditions of prolonged irradiation time, low initial dye concentration, high temperature, and acidic pH. The complete set of 3D response surface profiles is presented in Figure 10a–f. The corresponding 2D contour and 3D response surface plots for RB removal are shown in Figure 11 and Figure 12, respectively.

For RB, the contour plots indicate maximum degradation under prolonged irradiation time, intermediate initial dye concentration, higher pH, and lower catalyst dosage (Figure 11a–i). The same trends are confirmed by the 3D response surface plots (Figure 12a–i), demonstrating good agreement and consistency with the optimization model.

### 3.14. Diagnostic and Influencing Graphs of EB and RB RSM

Diagnostic and influencing plots are valuable tools for studying the efficacy of a model. These plots provide insights into the goodness of fit, normality of data distribution, and identification of potential abnormal runs or outliers. Several of these plots are shown in Figures S3 and S4. Figures S3a and S4a display the normal probability graphs of EB and RB, respectively. In these graphs, the points should ideally follow a straight line to indicate a normal distribution. It is observed that the points in both cases show slight deviations from the straight line, confirming the normal distribution of the data with minor irregularities. The externally standardized residuals versus predicted response values are depicted in Figures S3b and S4b for EB and RB, respectively. This plot helps estimate the deviation of the actual values’ standard deviation from the predicted values. In RSM, a control limit of ±3.94 and ±3.92 is suggested for EB and RB respectively, to detect abnormal runs. In the case of EB and RB degradation, no abnormal runs were detected, indicating the goodness of fit of the proposed model. Figures S3c and S4c show the residuals versus run number values. Typically, the values in this graph should be randomly scattered, but they should not exceed the suggested ±3.94 to ±3.92 range for EB and RB, proposed by RSM. In the case of EB and RB removal, the values are randomly scattered and fall within the range specified by RSM, demonstrating the validity of the proposed model. The graphs in Figures S3d and S4d represent the actual versus predicted values. Ideally, the points should be distributed around a straight line. If any points deviate significantly from the line, it indicates a problem that should be investigated. In the case of EB and RB degradation, all the points are well distributed around a straight line, confirming the good fit of the models. Based on these diagnostic and influencing plots, it can be concluded that the proposed models show a good fit. The normality of data distribution, absence of abnormal runs, random scattering of residuals, and the alignment of actual and predicted values indicate the reliability and effectiveness of the proposed model in describing the degradation of EB and RB dyes.

## 4. Conclusions

Cu-CoS_2_ photocatalyst was successfully employed for the photodegradation of both single and binary mixtures of cationic RB and anionic EB dyes under sunlight irradiation. The results demonstrated that the photocatalytic degradation efficiency is strongly influenced by several operational parameters, including dye concentration, irradiation time, pH, temperature, and catalyst dosage. The synthesized Cu-CoS_2_ nanoparticles were systematically characterized using FTIR, XRD, SEM, EDX, and UV-Vis techniques, which confirmed their structural, morphological, and compositional properties. XRD analysis revealed the crystalline phase of the photocatalyst, while SEM and EDX confirmed the morphology and elemental composition of Cu-CoS_2_, respectively.

The degradation behavior of both EB and RB dyes was significantly affected by solution pH, where EB showed higher degradation under acidic conditions (pH < 7), whereas RB exhibited enhanced degradation under alkaline conditions (pH > 7). An increase in initial dye concentration led to a decrease in photocatalytic efficiency. In contrast, degradation efficiency improved with increasing temperature, with maximum performance observed at 50 °C for the binary dye system. Catalyst dosage also played a key role, where degradation increased with increasing catalyst amount up to an optimum level, beyond which a decline was observed due to light scattering and reduced active site accessibility. Kinetic analysis revealed that the degradation rate constant of EB in single-dye systems was comparable to that in binary mixtures, indicating minimal interference from RB in the mixed system. However, RB exhibited higher degradation efficiency than EB in both single and binary systems. The photodegradation process followed second-order kinetics, which provided the best fit for the experimental data.

Future studies may focus on improving the visible-light harvesting efficiency of Cu-CoS_2_ through doping or heterojunction formation with other semiconductor materials. In addition, scaling up the photocatalytic system for real industrial wastewater treatment, along with long-term stability and reusability assessments, would be essential for practical environmental applications. Further investigations into mechanistic pathways and reactive species identification could also provide deeper insight into the degradation process.

## Figures and Tables

**Table 1:** Second-order kinetic equation parameters at different temperatures for EB and RB single and binary dye mixtures.

Sample	Temperature (°C)	R^2^	k_app_	Sample	Temperature °C	R^2^	k_app_
EB	30	0.98614	0.07208	EB	30	0.96366	0.12877
	40	0.95599	0.21744		40	0.94511	0.13762
	50	0.96986	0.37103		50	0.92942	1.35485
RB	30	0.81396	4.22772	RB	30	0.96366	1.35485
	40	0.83386	0.13741		40	0.95228	3.1571
	50	0.5438	1.82054		50	0.67458	8.14566

**Table 2:** Parameters of second-order kinetics for photocatalytic degradation of EB and RB dyes, single and binary mixture at different temperatures using Cu-CoS_2_.

Table		Second Order Kinetics				Activation Energy kJ/mol	Activation Energy kJ/mol
		R^2^	k_app_	R^2^	k_app_		
EB	30	0.98614	0.07208	0.96366	1.35485	66.84	72.40
	40	0.95599	0.21744	0.95228	3.1571		
	50	0.96986	0.37103	0.67458	8.14566		
RB	30	0.81396	4.22772	0.96366	0.12877	140.04	95.54
	40	0.83386	0.13741	0.94511	0.36711		
	50	0.5438	1.82054	0.92942	1.35485		

## Data Availability

The original contributions presented in this study are included in the article/Supplementary Materials. Further inquiries can be requested to the corresponding authors.

## Supplementary Materials

The following supporting information can be downloaded at: https://www.mdpi.com/article/10.3390/molecules31122152/s1.
