# Supplementary material for: Enhancing Photocatalytic Degradation Using Cu-CoS2 Nanoparticles for Solar-Driven Decolorization of Textile Dye Contaminants in Wastewater
Source: Molecules. 2026 Jun 18;31(12):2152. doi: 10.3390/molecules31122152 (PMC13305907; doi:10.3390/molecules31122152)
Supplement: Supplementary file 1 [file molecules-31-02152-s001.zip › molecules-4269633-supplementary.pdf]

Supplementary data

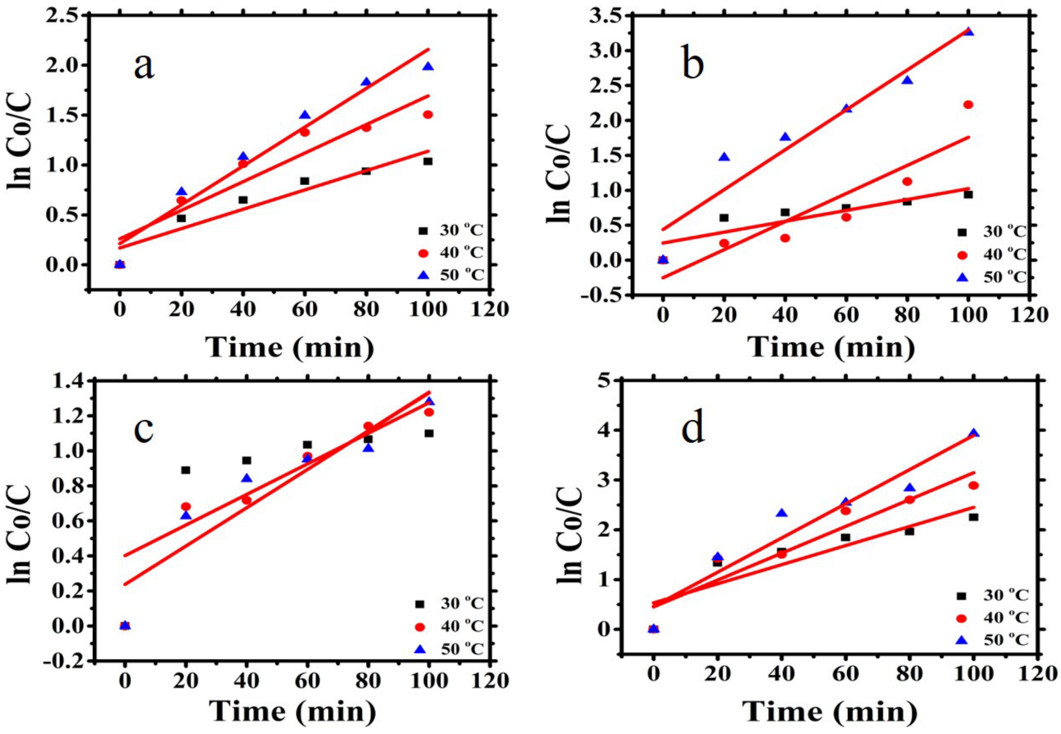

Figure: S1. Pseudo-first-order kinetic plots for the photocatalytic degradation of EB and RB dyes using Cu-CoS_2 nanoparticles under sunlight irradiation at different temperatures 30 °C, 40 °C, and 50 °C.

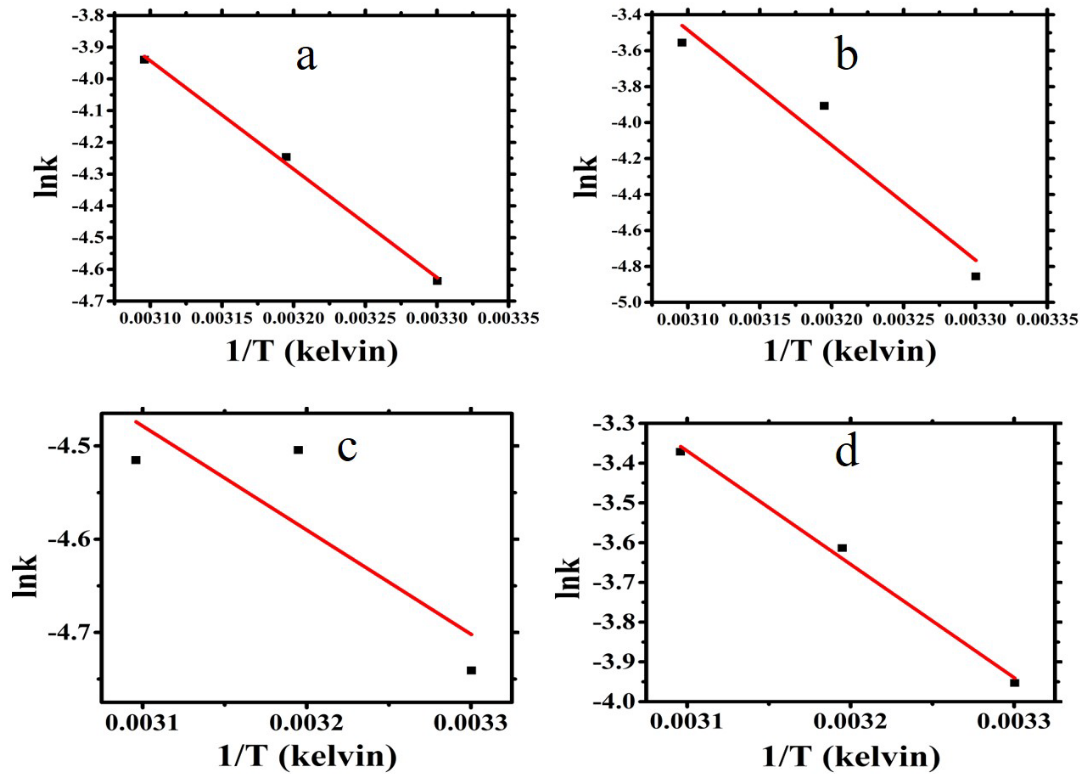

Figure: S2. Arrhenius plots (ln *k*versus1/T) for calculating the activation energy of the photocatalytic degradation of EB and RB dyes using Cu-CoS_2 nanoparticles

Table S1: First-order kinetic equation parameters at different temperatures for EB and RB single and binary dyes mixture.

Sample	Temp (°C)	R^2	k_{app}	Sample	Temp (°C)	R^2	k_{app}
EB	30	0.88645	0.00969	EB	30	0.51324	0.00873
	40	0.85526	0.01432		40	0.83774	0.01106
	50	0.94689	0.01945		50	0.83321	0.01094

RB	30	0.90732	0.00778	RB	30	0.76307	0.01919
	40	0.71163	0.0201		40	0.87699	0.02695
	50	0.80782	0.02856		50	0.90457	0.03434

Table S2: Parameters of first-order kinetics for photocatalytic degradation of single EB and RB dye at different temperatures using Cu-CoS_2

Temperature (°C)		1st Order Kinetics				Activation Energy	Activation Energy
		R^2	k_{app}	R^2	k_{app}	Kj/mol	Kj/mol
EB	30	0.94689	0.00969	0.00873	0.00873		
	40	0.85526	0.01432	0.01106	0.01106	28.35	9.28
	50	0.88645	0.01945	0.01094	0.01094		
RB	30	0.90732	0.00778	0.01919	0.01919		
	40	0.71163	0.0201	0.02695	0.02695	53.11	23.69
	50	0.80782	0.02856	0.03434	0.03434		

Table S3. ANOVA for Reduced Quadratic model EB degradation

Response 1: Degradation

Source	Sum of Squares	df	Mean Square	F-value	p-value	
**Model**	4600.07	18	255.56	183.44	< 0.0001	significant
A-Time	28.77	1	28.77	20.65	0.0004	
B-Dye Concentration	75.59	1	75.59	54.26	< 0.0001	
C-Cu-CoS2 dose	36.86	1	36.86	26.46	0.0001	
D-pH	260.96	1	260.96	187.32	< 0.0001	
E-Temprature	2.65	1	2.65	1.90	0.1884	
AB	7.86	1	7.86	5.64	0.0313	
AD	218.87	1	218.87	157.11	< 0.0001	
AE	105.24	1	105.24	75.54	< 0.0001	
BC	3.29	1	3.29	2.36	0.1454	
BE	8.36	1	8.36	6.00	0.0270	
CD	35.31	1	35.31	25.34	0.0001	
CE	74.62	1	74.62	53.56	< 0.0001	
DE	78.01	1	78.01	56.00	< 0.0001	
A^2	68.52	1	68.52	49.18	< 0.0001	
B^2	22.86	1	22.86	16.41	0.0010	
C^2	6.56	1	6.56	4.71	0.0465	
D^2	74.97	1	74.97	53.82	< 0.0001	

E^2	52.93	1	52.93	37.99	< 0.0001	
**Residual**	20.90	15	1.39			
Lack of Fit	10.17	8	1.27	0.8302	0.6036	not significant
Pure Error	10.72	7	1.53			
**Cor Total**	4620.97	33				

**Standard Deviation**	1.18
**Mean**	84.59
**Coefficient of variance**	1.40
**R^2**	0.9955
**Adjusted R^2**	0.9901
**Adequate Precision**	54.1800

Table S4. ANOVA for Quadratic model RB degradation

Source	Sum of Squares	df	Mean Square	F-value	p-value	
**Model**	9952.44	20	497.62	100.00	< 0.0001	significant
A-Time	1022.76	1	1022.76	205.53	< 0.0001	
B-Dye Concentration	160.09	1	160.09	32.17	< 0.0001	
C-Cu-CoS2 dose	3.55	1	3.55	0.7129	0.4109	
D-pH	1127.62	1	1127.62	226.60	< 0.0001	
E-Temprature	44.08	1	44.08	8.86	0.0089	
AB	248.28	1	248.28	49.89	< 0.0001	

AC	123.95	1	123.95	24.91	0.0001	
AD	385.31	1	385.31	77.43	< 0.0001	
AE	211.30	1	211.30	42.46	< 0.0001	
BC	210.08	1	210.08	42.22	< 0.0001	
BD	28.54	1	28.54	5.73	0.0292	
BE	210.68	1	210.68	42.34	< 0.0001	
CD	125.32	1	125.32	25.18	0.0001	
CE	255.26	1	255.26	51.30	< 0.0001	
DE	0.7912	1	0.7912	0.1590	0.6954	
A^2	555.23	1	555.23	111.58	< 0.0001	
B^2	96.18	1	96.18	19.33	0.0005	
C^2	691.08	1	691.08	138.88	< 0.0001	
D^2	73.78	1	73.78	14.83	0.0014	
E^2	2.35	1	2.35	0.4724	0.5017	
**Residual**	79.62	16	4.98			
Lack of Fit	55.22	7	7.89	2.91	0.0692	not significant
Pure Error	24.40	9	2.71			

**Standard Deviation**	2.23
**Mean**	75.69
**Coefficient of variance**	2.95
**R^2**	0.9921

**Adjusted R^2**	0.9821
**Predicted R^2**	0.7635
**Adequate Precision**	37.6990

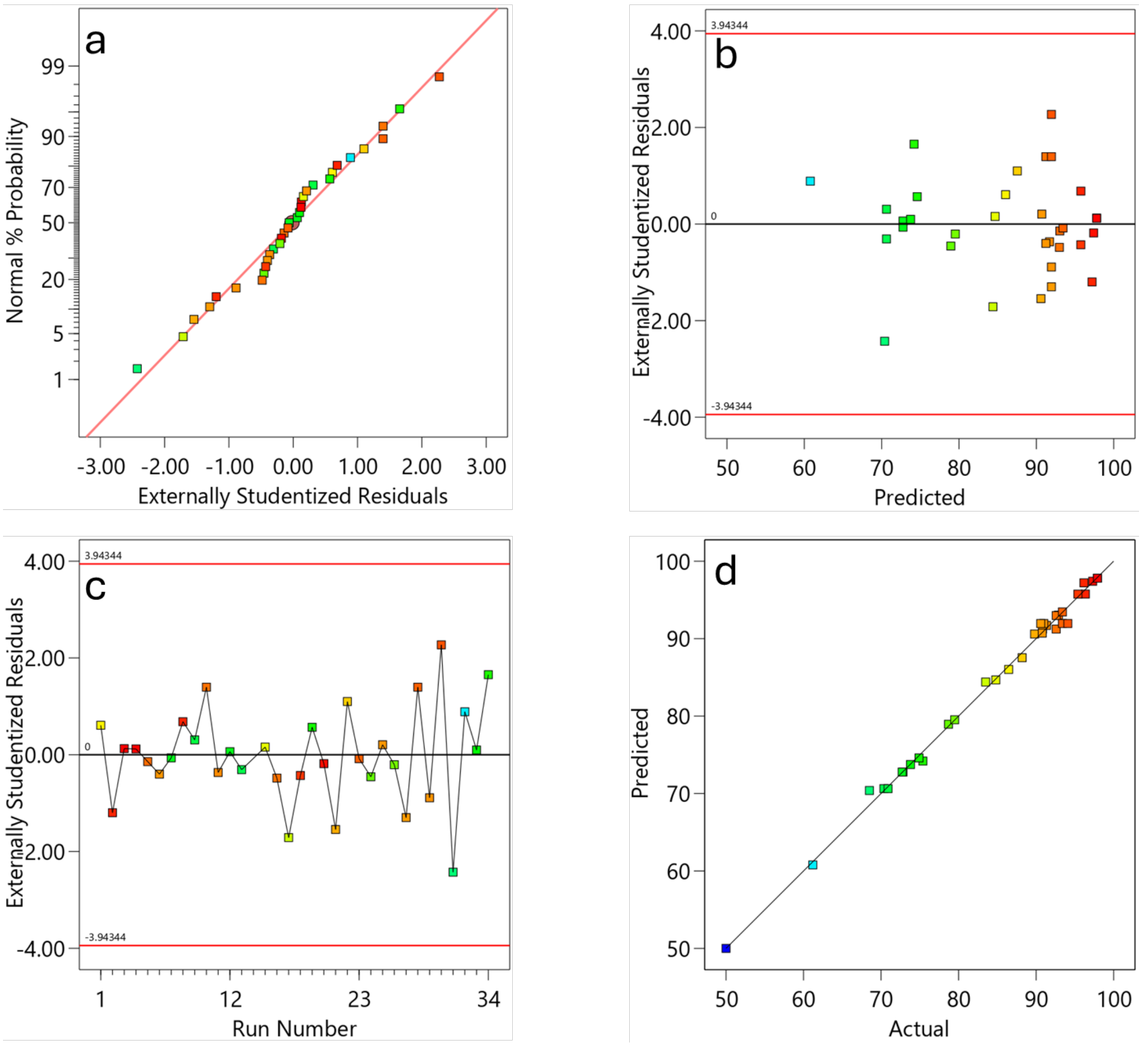

Figure S3. Diagnostic and residual influence evaluation plots for the validated Response Surface Methodology (RSM) reduced quadratic model optimizing EB photocatalytic degradation efficiency (%). **(a)** Normal probability plot of externally studentized residuals, where data points closely follow the diagonal reference line, confirming that residuals are approximately normally distributed and that the model satisfies the fundamental statistical

assumption of normality. **(b)** Externally studentized residuals plotted against predicted degradation efficiency values, revealing no systematic funnel-shaped pattern or trend, which confirms homoscedasticity (constant error variance) across the entire experimental response range (~50-100%). All residuals fall within the ±3.50 threshold limits (red boundary lines), indicating no statistically influential outliers. **(c)** Externally studentized residuals plotted against sequential run number (1-34), demonstrating the absence of any time-dependent drift, autocorrelation, or lurking variables introduced during experimental execution, residuals scatter randomly within the ±3.50 control limits, validating experimental randomization. **(d)** Predicted versus actual degradation efficiency (%) parity plot, where experimental data points cluster tightly along the 45° diagonal reference line across the full response range (~50-100%), confirming excellent agreement between model-predicted and experimentally observed values and validating the predictive accuracy and goodness-of-fit of the reduced quadratic RSM framework.

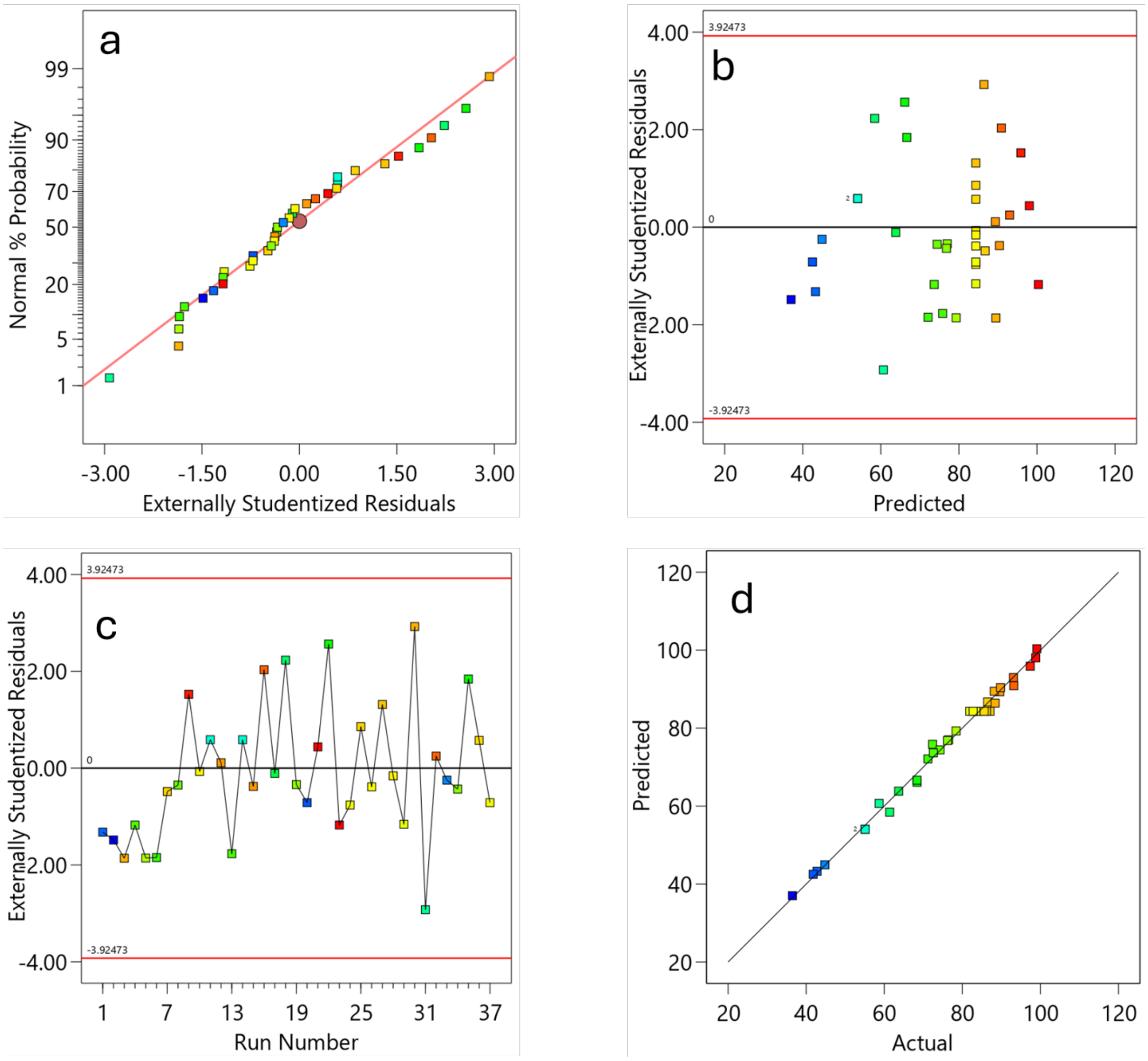

Figure S4. Diagnostic and residual influence evaluation plots for the validated Response Surface Methodology (RSM) full quadratic model optimizing RB photocatalytic degradation efficiency (%)**. (a)** Normal probability plot of externally studentized residuals, where data points align closely along the straight diagonal reference line across the probability range (1–99%), confirming that model residuals follow a normal distribution, validating the statistical integrity of the quadratic model and supporting reliable hypothesis testing within the ANOVA framework. **(b)** Externally studentized residuals versus predicted degradation efficiency values (~20-120%), showing random, structureless scatter around the zero baseline with no discernible heteroscedastic pattern or curvature trend. All residuals are bounded within the

±3.50 red limit lines, confirming the absence of outliers and equal error variance throughout the modeled response space. **(c)** Externally studentized residuals plotted sequentially against run number (1-37), displaying random oscillation around zero without any systematic trend, cyclical pattern, or progressive drift, confirming that experimental runs were properly randomized and that no time-order bias or environmental confounding influenced the response measurements. **(d)** Predicted versus actual degradation efficiency (%) parity plot across the broad experimental range (~20-120%), demonstrating that model-predicted values align closely with the 45° ideal diagonal, particularly at mid-to-high efficiency values, with minor scatter at lower degradation levels, collectively confirming the strong predictive capability, robustness, and reliability of the full quadratic RSM model for RhB removal optimization.
